# Persian adaptation and validation of the user version of The Mobile Application Rating Scale (uMARS)

**DOI:** 10.1371/journal.pone.0320349

**Published:** 2025-04-02

**Authors:** Zeinab Kohzadi, Shahabedin Rahmatizadeh, Zahra Kohzadi, Saeideh Valizadeh-Haghi

**Affiliations:** 1 Department of Health Information Technology and Management, School of Allied Medical Sciences, Student Research Committee, Shahid Beheshti University of Medical Sciences, Tehran, Iran,; 2 Department of Health Information Technology and Management, School of Allied Medical Sciences, Shahid Beheshti University of Medical Sciences, Tehran, Iran,; 3 Department of Health Information Management and Technology, Kashan University of Medical Sciences, Kashan, Iran,; 4 Department of Medical Library and Information Science, School of Allied Medical Sciences, Shahid Beheshti University of Medical Sciences, Tehran, Iran; Iran University of Medical Sciences, IRAN, ISLAMIC REPUBLIC OF

## Abstract

**Objective:**

Health applications (apps) enable patients to make decisions regarding their health utilizing digitally-enabled processes for care, expand accessibility to healthcare service delivery, and raise public awareness of health. In many languages, there is still a lack of an instrument that assesses how patients perceive apps. This study aims to adapt and validate the User Version of the Mobile Application Rating Scale (uMARS) in Persian and evaluate the overall quality of the Persian meditation apps.

**Methods:**

This cross-sectional study population comprises 86 healthcare workers in a health center in the west of Iran. The sample size was determined using Cochran’s formula. First, the uMARS was translated into Persian. Then, validity was assessed using the Content Validity Ratio (CVR), Content Validity Index (CVI), and face validity. Cronbach’s alpha and Intraclass Correlation Coefficients (ICC) were used to assess reliability. Among the 124 meditation apps, Aramia met the criteria included in the study and was selected to evaluate objective quality, subjective quality, and perceived impact.

**Results:**

The majority of the participants were female (82.55%). More than half of the participants had a bachelor’s degree (58.14%). The CVR, CVI, ICC, and Cronbach’s alpha values were obtained as 0.79. 0.90, 0.93, and 0.86, respectively. These Findings revealed that the Persian version of the questionnaire has sufficient reliability and validity. Among the three subscales, perceived impact received the highest mean score (3.96 ± 0.37), and a total score of 3.74 ± 1.04 was obtained.

**Conclusions:**

A Persian version of uMARS is a valid and reliable tool for evaluating the quality of mobile health apps from the user’s point of view during the development and testing process and improving the app’s quality.

## Introduction

Progress in mobile phone technology over the past two decades has allowed individuals to access health information anywhere due to widespread internet connectivity. Moreover, mobile phones can be employed by public health and healthcare organizations to share health information and provide consistent, unbiased support [1]. The demand for more accurate and tailored medicine, the unsustainable nature of current healthcare expenditure, and the rapid and continuous growth of wireless connectivity have made “mobile health” increasingly popular in recent years [2]. The term “mobile health” or “mHealth” refers to the use of portable electronic devices, such as smartphones and tablets, to support medical services and improve patient outcomes [3]. mHealth solutions are widely used in public health and healthcare services, where they are valued for their simplicity, broad reach, and widespread acceptance [4]. Indeed, interest in digital health technology is growing, and more than half of smartphone users have downloaded at least one health-related application (app) at some point [5]. Smartphone health apps help patients in many ways, including better self-management, increased engagement, convenience, financial savings, and improved provider-patient communication [6]. Through a variety of mechanisms, mobile health apps have the potential to improve patient outcomes significantly [7]. These apps can improve patient engagement, help patients with self-management, provide educational resources, enable remote monitoring, and promote treatment adherence [8,9]. Research indicates that mobile health apps can affect health outcomes by improving the quality of life, controlling clinical parameters, promoting medication adherence, managing symptoms, reducing complications, and contributing to overall well-being [10–12]. Users of health-related apps risk a range of potential problems, such as adverse health effects, inaccurate or misleading information, absence of accountability, inefficient healthcare, and privacy issues, if they are not evaluated and regulated properly [13,14].

However, the quality of mHealth apps could be improved. It is concerning that many health-related apps lack substantial evidence to support their claims [15]. Evaluating the quality and content of these apps is essential to ensure that both users and experts can trust their credibility and effectiveness [16]. The Mobile Application Rating Scale (MARS) is a tool used by experts to evaluate the quality of mHealth apps, and the user version of the Mobile Application Rating Scale (uMARS) is a simplified version of MARS that end-users can use to evaluate mHealth app quality. The uMARS tool is a practical and feasible means of assessing the quality of an app by a layperson with no specialized training [17,18]. Researchers can determine the aspects of health app quality that users have evaluated as positive and negative and highlight areas requiring further development [19]. In addition, the scale has helped increase engagement scores, usability, functionality, and perceived impact of health apps or the benefits of programs that use apps [20–22] In some studies, uMARS has evaluated the app’s quality. Bardus et al. evaluated the quality of mobile apps designed to help manage weight [23]. LeBeau et al. assessed the quality of mobile apps used by occupational therapists [24], and Adam et al. evaluated the quality of mobile apps that calculate prostate cancer risk [25]. Several versions of uMARS are available in various languages, all of which have been translated and verified [26–29]. However, this instrument has no translation or cultural adaptation into Persian. The Persian version of uMARS can be used by the Persian community to evaluate mobile apps. Therefore, this study aims to adapt and validate uMARS in Persian and evaluate the overall quality of the Persian meditation apps.

## Method

### Study Design and Participants

This study was a cross-sectional survey conducted in January 2024. The population studied consisted of healthcare workers in a health center in the city of Ilam, in the west of Iran. The sample size, determined using Cochran’s formula with a 95% confidence level, was calculated to be eighty-six individuals. The inclusion criteria were having at least three years of work experience and having an Android or iOS phone. Conversely, the exclusion criteria were having less than three years of experience and not having an Android phone.

This research has been approved by the Ethics Committee of Shahid Beheshti University of Medical Sciences (Ethics Code: IR.SBMU.RETECH.REC. 1402.458). In this research, verbal consent was obtained from the participants. Participants were given the Participant Information Sheet to read for obtaining their consent. Once the participants comprehensively considered the study and discussed it with researchers, they were allowed to ask any questions they may have. Once all questions were answered and the researcher was confident that the participant fully understood the study requirements, they asked if they would like to consent. If the participant agreed to proceed, the researcher obtained a witness unrelated to the study. A note was added to the participant’s record stating the date oral consent was obtained, the study’s details, and the researcher’s name and witness present during oral consent. Notably, participants were allowed to withdraw from the study at any time. Furthermore, no identifiable information was collected from the participants.

During the ethics review, the committee decided that a signed consent form was unnecessary. Hence, the consent form was read and explained to respondents during data collection instead of requiring written consent. The research objectives were verbally explained to all participants by two research team members. After addressing their questions, if they expressed interest, the questionnaire was provided to them for completion. Additionally, at the beginning of the questionnaire, the research objectives, methodology, and confidentiality were explained again, and participants were allowed to withdraw from the study at any time. No identifiable information was collected from the participants. The confidentiality of the participant’s responses was also ensured.

### Instruments

The study used a two-section questionnaire. The first section is about the demographic characteristics of the participants (gender, age, education, and field of education), and the second section includes the uMARS.

uMARS tool is a multidimensional tool used to evaluate the overall quality of mobile health apps developed and validated by Stoyanov et al. in 2016 [17]. The uMARS has three key dimensions: objective quality, subjective quality, and perceived impact. Objective quality relates to the technical aspects of the product, including usability, reliability, and functionality. Subjective quality is based on users’ thoughts and feelings, such as whether the product is enjoyable, easy to use, and meets their needs. Perceived impact measures how much a product positively affects the user’s learning, such as improving knowledge, skills, or motivation. Twenty items in uMARS evaluate the objective and subjective quality of apps. They are scored on a 5-point Likert Scale, with one meaning “poor” and five meaning “excellent” quality. Additionally, an extra option exists to select “Not applicable” for items 13 –16. The mean of the four dimensions’ scores is used for calculating the objective quality score: engagement (items 1–5), functionality (items 6–9), aesthetics (items 10–12), and information (items 13–16). The mean of four subjective items (17–20) obtains a subjective quality score. The last subscale of uMARS consists of six items that are rated on a 5-point Likert Scale from one (strongly disagree) to five (strongly agree) to evaluate how the user perceives the app’s impact on their awareness, knowledge, attitudes, intention to change, help-seeking, and a probability of changing the target health behavior [17]. The Interclass Correlation Coefficients (ICC) in the uMARS version (0.66 and 0.70 in two distinct periods) are at appropriate levels. The overall score for each section is calculated by dividing the sum of the scores for each question by the total number of questions in the section. The total uMARS score is calculated by dividing the sum of the scores for each section by the total number of questions [17]. In this study, a score below three is considered poor, three to four is considered moderate, and above four is considered good.

### Cross-cultural adaptation and translation process

After obtaining permission from the original questionnaire owners, two Persian (Farsi) language researchers fluent in Persian and English with expertise in health and Information Technology (IT) terminology independently translated the questionnaire into Persian. To reach an agreement, the translators checked the translations for discrepancies and compiled the preliminary version in Persian. The entire Persian version was translated into English by a native English speaker who was fluent in Persian and blind to the original English terminology and phrases in the questionnaire. The English back-translated version has been checked against the original by a team of experts to ensure no conceptual or cross-cultural inconsistencies.

### Statistical analysis

The study evaluated the content and face validity through quantitative and qualitative methods. Qualitative face validity was determined through a 12-expert panel of medical informatics and health information management. The purpose was to identify the difficulty, inconsistency, and ambiguity in phrases or inadequacies in the meanings of words. The experts provided their opinions in the form of minor changes to the questionnaire. The Impact score of the question was calculated to determine quantitative face validity. First, a five-point Likert scale was used for each tool item: completely agree (five points) to completely disagree(one point). Then, the questionnaire was given to 15 healthcare workers to determine its validity. After the target group had completed the questionnaire, we calculated the face validity using the following formula:


impact score=frequency×importance (1)

An acceptable impact score for this study is at least 1.5 [30].

For the qualitative evaluation of content validity, experts were requested to provide their opinions after a detailed instrument study. Notably, the qualitative evaluation of content validity considers grammar, appropriate wording, question meaning, and the placement of questions in their proper place. After gathering expert opinions, the necessary changes were applied to the tool. The Content Validity Ratio (CVR) was used to quantitatively evaluate the validity of the content and ensure that the most important and accurate content (i.e., necessary questions) was selected. Additionally, the Content Validity Index (CVI) was used to ensure that the tool’s questions were designed in the best way possible to measure the content. experts were asked to evaluate each of the tool’s questions to determine the content validity of the questionnaire. The experts were asked to indicate whether each question was “necessary,” “not necessary, but useful,” or “not necessary to answer.” The answers were then calculated using the CVR formula and adapted to Lawshe’s table. Only scores above 0.56 were accepted [31]. Then, the questionnaire was given to the experts for calculating CVI, and they were asked to evaluate three criteria of relevance, simplicity, and clarity based on a four-point Likert scale (for example, one: irrelevant, two: Somewhat relevant, three: relevant, and four: completely relevant). The number of experts who chose options three and four was divided by the total number of experts to calculate the CVI score. Scores above 0.79 were considered acceptable [32,33].

Cronbach’s alpha was used to determine the degree of internal consistency of the uMARS subscales and overall score. A Cronbach’s alpha value above 0.90 was interpreted as excellent, 0.80-0.89 as good, 0.70-0.79 as acceptable, 0.60-0.69 as questionable, 0.50-0.59 as poor, and < 0.50 as unacceptable [34].

The uMARS subscales and total scores were tested for test-retest reliabilities. ICC values less than 0.50, 0.50-0.75, 0.75-0.90, and more than 0.90 are regarded as low, moderate, good, and excellent reliability, respectively [35]. After two weeks, a retest was completed by twenty-four randomly selected healthcare workers.

The questionnaire in paper format was distributed among the study participants. The data were analyzed using SPSS version 18.0 (SPSS Inc, Chicago, IL, USA).

### App selection process

Following the translation into Persian, a thorough app search was conducted on January 2024, utilizing Google Play Store (Android), App Store (iOS), and Cafe Bazaar, the most well-known app store in Iran. Keywords such as “meditation” and “meditation Persian” were searched in these stores. A mobile app was selected based on the following inclusion criteria: availability in Persian, relevance to meditation, free availability, achieving a user rating of over four stars out of five, maintaining a minimum of twenty thousand installations, and being updated within the past six months; the data was recorded in a Microsoft Excel spreadsheet for subsequent analysis using descriptive frequency statistics. During the selection process, emphasis was placed on software well-supported by its company, regularly updated within the past year to ensure ongoing improvement, widely installed to showcase its popularity and user familiarity, and consistently maintains high user ratings to reflect increased satisfaction and acceptance.

Ultimately, the Aramia App was selected. The Aramia Persian App specializes in meditation, yoga, mindfulness, and sub-categories such as relaxation, concentration, peaceful sleep, pain control, and stress management. It also includes breathing exercises and night-time relaxation stories. Additionally, the app offers over 15 meditation courses designed to achieve specific goals.

## Results

Table 1 presents the participants’ demographic information. The female participants constituted the majority (82.55%) of the total participants. A significant proportion (58.14%) of the participants held a bachelor’s degree. Moreover, most participants’ education field was public health (60.47%). Notably, Table 1 did not have any missing responses.

**Table 1:** Demographic characteristics of participants (n = 86).

Variables	N (%)	Mean±SD
Gender		
Female	71(82.55)	
Male	15(17.45)	
Age		36.29±8.71
Education		
Bachelor	50 (58.14)	
Master	29(33.72)	
Ph. D	7 (8.14)	
Field of Education		
Public health	52(60.47)	
Midwife	21(24.42)	
Occupational health	5(5.81)	
Environmental Health	5(5.81)	
Nutritionist	3 (3.49)	

**SD:** Standard Deviation

### Validity and reliability

The questionnaire included questions with a face content validity score equal to or greater than 1.5. Table 2 shows the test-retest reliability (ICC) results, CVI, and CVR for uMARS, which were 0.93, 0.90, and 0.79, respectively. Given that CVR, CVI, and ICC are more than 0.75, 0.79, and 0.56, respectively, the Persian version of the questionnaire has acceptable reliability and validity.

**Table 2:** Content validity and Reliability Statistics for the uMARS.

Subscale	Item	CVR	CVI	ICC	95% CI
A - engagement	A1 - entertainment	0.69	0.90	0.85	(0.80,0.89)
	A2 – interest	0.69	0.87	0.91	(0.85, 0.96)
	A3 - customization	1.00	0.97	0.95	(0.91, 0.98)
	A4 - interactivity	0.69	0.87	0.95	(0.91, 0.98)
	A5 - target group	1.00	0.97	0.86	(0.81, 0.90)
	Total	0.81	0.91	0.92	(0.86, 0.97)
B - functionality	B6 - performance	1.00	0.90	0.98	(0.95, 1.00)
	B7 - ease of use	0.85	0.92	0.96	(0.92, 0.99)
	B8 – navigation	0.69	0.90	0.88	(0.82, 0.93)
	B9 - gestural design	0.85	0.87	0.92	(0.86, 0.97)
	Total	0.84	0.89	0.94	(0.89, 0.98)
C - esthetics	C10 – layout	0.69	0.97	0.91	(0.85, 0.96)
	C11 – graphics	0.85	0.92	0.92	(0.86, 0.97)
	C12 - Visual Appeal	0.69	0.90	0.87	(0.80, 0.93)
	Total	0.74	0.93	0.91	(0.85, 0.96)
D - information	D13 - quality of information	0.69	0.92	0.95	(0.91, 0.98)
	D14 - quantity of information	0.69	0.90	0.84	(0.79, 0.88)
	D15 - visual information	0.54	0.92	0.97	(0.94, 0.99)
	D16 - credibility of source	0.85	0.85	0.86	(0.81, 0.90)
	Total	0.69	0.89	0.93	(0.88, 0.97)
Objective quality	Total	0.77	0.90	0.93	(0.88, 0.97)
E - subjective quality	E17-recommendation to others	0.85	0.97	0.94	(0.89, 0.98)
	E18 - use and relevance	0.69	0.97	0.91	(0.85, 0.96)
	E19 – payment	0.85	0.85	0.96	(0.92, 0.99)
	E20 - overall rating	0.85	0.87	0.83	(0.77, 0.88)
	Total	0.81	0.91	0.91	(0.85, 0.96)
Total		0.79	0.90	0.93	(0.88, 0.97)

ICC intraclass correlation coefficient, CVR Content Validity Ratio, CVI Content Validity Index, CI Confidence Interval uMARS the User Version of the Mobile Application Rating Scale.

Table 3 shows Cronbach’s alpha values obtained for the subscales. The highest-scoring subscale was objective quality, with a score of 0.84. The information item quality subscale followed closely, scoring 0.74. The overall Cronbach’s alpha was 0.86, indicating good reliability as it exceeded the acceptable threshold of 0.7.

**Table 3:** Cronbach’s alpha of uMARS subscales.

Subscale	α
A - engagement	0.72
B - functionality	0.72
C - esthetics	0.73
D - information	0.74
Objective quality	0.84
E - subjective quality	0.73
Total	0.86

**α** Cronbach’s alpha, **uMARS** the User Version of the Mobile Application Rating Scale.

### App assessment

Table 4 shows the mean scores for the Aramia App. Among the three subscales (Objective quality, subjective quality, perceived impact), perceived impact received the highest mean score (3.96 ± 0.37), and subjective quality received the lowest mean score (3.49 ± 0.34). The overall uMARS score for Aramia was 3.74 ± 1.04.

**Table 4:** The mean scores for each subscale and item of the uMARS.

Subscale	Item	Mean Score ± SD
A - engagement	A1 - entertainment	3.56 ± 0.75
	A2 - interest	3.95 ± 1.08
	A3 - customization	2.91 ± 1.18
	A4 - interactivity	3.06 ± 1.11
	A5 - target group	3.41 ± 0.89
	Total	**3.38 ± 0.36**
B - functionality	B6 - performance	4.06 ± 0.97
	B7 - ease of use	3.62 ± 0.87
	B8 - navigation	4.08 ± 1.00
	B9 - gestural design	4.04 ± 0.95
	Total	**3.95 ± 0.19**
C - esthetics	C10 - layout	3.95 ± 1.01
	C11 - graphics	4.02 ± 1.02
	C12 - visual Appeal	3.30 ± 1.05
	Total	**3.75 ± 0.32**
D - information	D13 - quality of information	3.95 ± 1.04
	D14 - quantity of information	3.40 ± 0.86
	D15 - visual information	4.06 ± 1.02
	D16 - credibility of source	4.09 ± 0.97
	Total	**3.88 ± 0.27**
Objective quality		**3.74 ± 0.28**
E - subjective quality	E17-recommendation to others	4.08 ± 0.93
	E18 - use and relevance	3.34 ± 0.89
	E19 - payment	3.36 ± 1.02
	E20 - overall rating	3.19 ± 1.02
	Total	**3.49 ± 0.34**
F - perceived impact	F1 - awareness	4.09 ± 0.84
	F2 – knowledge	4.16 ± 0.83
	F3 – attitudes	4.33 ± 0.82
	F4 - intention to change	4.27 ± 0.78
	F5 - help-seeking	3.60 ± 0.85
	F6 - behavior change	3.31 ± 0.91
	Total	**3.96 ± 0.37**
Total score		**3.74 ± 1.04**

## Discussion

The uMARS is a valuable tool for researchers and app developers to identify highly valued and praised components and opportunities for further improvement. It also aids healthcare professionals in recommending quality mobile apps to their patients [17,26,36]. This study presents the first translation and adaptation of the uMARS scale into Persian.

The results indicate that the Persian version has acceptable validity and reliability, and all subscales of objective and subjective quality showed adequate internal consistency and test-retest reliability. This finding is consistent with other comparable studies and the original English version [17,26–28]. The present study’s findings showed that the participants rated the Aramia app moderately. In this app, the highest score was received by the perceived impact subscale since Aramia’s app increased awareness, knowledge, attitudes, and intention to change in meditation.

The overall uMARS score for Aramia was 3.74 which is comparable to the findings of the similar studies [37]. It is also found that the Aramia app has received a poor score in the customization section. This finding is similar to the findings of the study by Lambrecht et al. evaluating the quality of a mobile app to support patients with rheumatic diseases, the customization item received the lowest score [37]. These findings show that mobile app developers need to consider customization to increase their users’ satisfaction with that specific app. Occupational stress and burnout affect every professional, but healthcare workers are most at risk [38,39]. Seemingly, meditation practices facilitated by the Aramia app can improve their mental health and overall well-being, leading to better focus, attention, decision-making, and patient care. By practicing mindfulness, healthcare workers can enhance their ability to stay present and focused in demanding situations. In addition, meditation applications offer healthcare workers a valuable resource for stress management, improved mental health, and overall well-being. Healthcare workers can boost their personal and professional lives by incorporating mindfulness practices into their daily routines using these apps.

The participants in this study were healthcare workers, but findings from other studies might differ based on participant types. Therefore, we recommend conducting further research on a larger scale with diverse samples to validate the Persian version of the uMARS across various health program categories. Additionally, while the uMARS focuses on assessing the quality and functionality of mobile health apps, it needs to precisely evaluate the credibility of the information these apps provide. Including an assessment of information credibility in future studies would be highly valuable.

## Conclusion

The study confirmed that the Persian version of the uMARS (PuMARS) is as valid and reliable regarding cross-cultural congruence as the original. This study’s findings indicate that the PuMARS holds significant promise for expansion. Both researchers and application developers can utilize this tool to gather feedback from end-users and pinpoint valuable elements and prospects for enhancement, thus guaranteeing enhanced quality and pertinence of mHealth. By employing the PuMARS scale, developers can identify areas that require improvement and make well-informed choices to optimize their applications for improved user interaction and heightened user satisfaction.

## Supporting information

Data Research data. (XLSX)
